# Differences in Kinetics during One- and Two-Leg Hang Power Clean

**DOI:** 10.3390/sports9040045

**Published:** 2021-03-27

**Authors:** Ryohei Hayashi, Takuya Yoshida, Yasushi Kariyama

**Affiliations:** 1 Faculty of Education, Gifu University, Gifu 501-1193, Japan; 2 Faculty of Health and Sport Sciences, University of Tsukuba, Tsukuba 305-8574, Japan; yoshida.takuya.gm@u.tsukuba.ac.jp; 3 Faculty of Sport Sciences, Yamanashi Gakuin University, Kofu 400-8575, Japan; y-kariyama@ygu.ac.jp

**Keywords:** weightlifting exercise, power training, resistance training, bilateral deficit

## Abstract

The purpose of this study was to quantify the kinetics per leg during the one- and two-leg hang power clean using various loads. Nine male track and field athletes performed the one- and two-leg hang power clean on a force platform. The estimated one-repetition maximum was used for the one-leg hang power clean (OHPC), and the one-repetition maximum was used for the two-leg hang power clean (THPC). The loads used were 30%, 60%, and 90% during both trials. We calculated peak power, peak force, and peak rate of force development during the pull phase from the force-time data. The peak power and the peak force for all loads during the OHPC were statistically greater than during the THPC. The peak rates of force development at 60% and 90% during the OHPC were statistically greater than during the THPC. Additionally, the peak power at 90% was significantly less than at 60% during the THPC. These findings suggest that the OHPC at loads of 60% and 90% is a weightlifting exercise that exhibits greater explosive force and power development characteristics than the THPC.

## 1. Introduction

Muscular power is the main factor that determines dynamic sports performance; many sports events require the athlete to exert a large amount of force in a short period of time [1,2]. Sprinting, agility, and jumping performance are improved by enhancing the power output of the lower limbs [3]. The ability to exert a high level of muscular power is an important component for improving performance in many sports events [4]. Therefore, many athletes focus on improving power output in the lower extremities.

Weightlifting exercises have been used as a training method to improve the power output of the lower limbs [1]; consequently, many sport movements have incorporated weightlifting movements such as the triple extension [5]. The hang power clean (HPC) is performed by many athletes for weightlifting exercises. Several studies indicate that peak power occurs at a load of 65–80% one-repetition maximum (1RM) during the HPC [1,4,6]. Furthermore, the 1RM of the HPC relates to the jump height of a countermovement jump (r = 0.41), sprint performance (r = −0.58), and agility (r = −0.41) [7].

Many sports movements, such as running, kicking, changing running direction, and jumping, involve phases of receiving a load on one leg [8]. Due to the bilateral deficit [9], it has been shown that the vertical ground reaction force per leg is greater for one-leg movements than for two-leg movements in jumping [10] and squat exercises [8,11]. Although Bosch [12] reported that the increased energy-transporting ability of the stance leg during a one-leg HPC (OHPC) develops greater overload, the kinetic characteristics during an OHPC remain unclear. Based on the bilateral deficit theory, the kinetics during the OHPC are considered greater than during the two-leg HPC (THPC). In lower limb kinetics, hip abductors are important for one-leg movements such as sprint running [13] and jumping [14]. These are also utilized in resistance training when performed with one leg [15]. Due to similarities in the kinetic characteristics of the lower limbs between sports performance and one-leg resistance training, the OHPC may be used as specific training for sport events that have phases of explosive one-leg movements. Consequently, the difference in the kinetics between the OHPC and THPC should be investigated for a better understanding of the characteristics of power output during the HPC.

The magnitudes of peak power, peak force, and peak rate of force development (RFD) [16] are different between loads during a THPC [1,4,6] and other pull movements [17,18,19,20]. Considering the bilateral deficit theory, the kinetic characteristics of THPC and OHPC may differ between loads. Thus, it is necessary to use various loads when comparing the kinetics of the OHPC and THPC.

Therefore, the purpose of this study was to quantify peak power, peak force, and peak RFD per leg during the OHPC and THPC using various loads. It was hypothesized that the kinetics during the OHPC would be greater than during the THPC at all loads, and the OHPC should be considered as a weightlifting exercise with greater peak power, peak force, and peak RFD than the THPC.

## 2. Materials and Methods

### 2.1. Participants

Nine male track and field athletes (mean ± SD age, 21.3 ± 2.2 years; height, 1.75 ± 0.05 m; mass, 67.4 ± 3.8 kg; 1RM THPC, 96.5 ± 8.18 kg; and relative 1RM THPC, 1.44 ± 0.16 kg·kg^−1^) participated. All participants were members of the university track and field team, and were familiar with the experimental trials. They had at least 5 years of resistance training and used both THPC and OHPC in their regular resistance training. The exclusion criteria were the following: use of medication affecting exercise capacity, or orthopedic limitations. All participants were over the age of 18 years and were informed of the benefits and risks of the investigation prior to signing a written informed consent form. The study was approved by the University of Tsukuba Research Ethics Committee (certificate number: 27-121).

### 2.2. Design and Procedures

All participants performed a 5-min warm-up of light cycling followed by a series of 10-min dynamic stretches. The 5 min of light cycling was performed using a bicycle ergometer (POWER MAX VIII, Konami Sports Co., Tokyo, Japan), and the loads were equalized among the participants. To determine 1RM of the THPC before the test session, a submaximal THPC using 30%, 50%, 70%, and 90% of each participant’s latest 1RM was performed, and the weight was gradually increased by 2.5–5.0 kg until the 1RM was established. A successful trial was recorded when a participant maintained a static upright posture after catching the barbell. The OHPC trial was conducted using half the barbell weight of the THPC 1RM [11,21]. We attempted to measure 1RM of the OHPC before the test session, although it was difficult for the participants to perform the catching motion in the same posture as in the THPC when the weight approached 1RM (for example, large lateral bending motion of the trunk). However, it was possible to set the same motion level for the THPC and OHPC by using the estimated 1RM for OHPC. In addition, the participants avoided the risk of injury while measuring 1RM for the OHPC. Therefore, the estimated 1RM was used for the OHPC in this study.

The test session was performed 2–4 days after the 1RM measurement. All participants performed a standardized warm-up and 1 warm-up set of three repetitions of the OHPC and THPC, at loads of 30% and 60% estimated 1RM, or 1RM. After the warm-up, participants performed two trials each of the THPCs at loads of 30%, 60%, and 90% 1RM. Following 5 min of rest, participants performed two trials each of OHPC at half loads of 30%, 60%, and 90% of two-leg 1RM. Participants were given 1 min of rest between trials, and at least 3 min of rest between each load for both HPCs. The THPC movements were performed using the technique described in previous studies [1,4,6]. Participants started with the barbell at the mid-thigh, lowered it to a position just above the knee, and returned it to the mid-thigh position. Participants then performed the pull movement with a triple extension of the hip, knee, and ankle, and by shrugging their shoulders. The barbell was lifted upward with maximal effort and caught on the shoulders in a semi-squat position.

**Figure 1:**
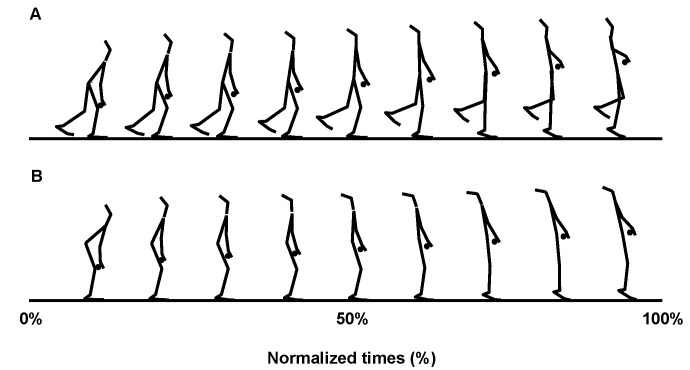
Typical stick images representing the pull phase from a sagittal view. (**A**): One-leg HPC. (**B**): Two-leg HPC. HPC, hang power clean.

The OHPC movement was conducted with participants standing on the dominant leg in the initial position with the same pull and catch technique used for the THPC. The dominant leg was defined as the leg used for jumping. To clarify the kinetic characteristics utilized during the OHPC by using the maximum effort trials, movement of the free leg was not restricted in the OHPC. Figure 1 shows the pull phase movements during the OHPC and THPC.

To set the initial posture during both HPCs, the knee joint angle of the dominant leg was monitored with a goniometer (SG150, DKH Co., Tokyo, Japan) to ensure that the position was accurately reproduced between both HPCs. The knee joint angle of the OHPC was defined as the knee joint angle of the THPC ± 5°. It has been shown that the pull motion does not significantly change the kinetics even if the knee and trunk angles are different [22]. For this reason, we did not use a specific knee angle. In addition, the height of participants’ line of sight in the initial posture were all equalized in the trial. A successful trial of both HPCs was defined as for when performing the 1RM of the THPC.

### 2.3. Measures

The three-dimensional coordinates of 47 retro-reflective markers (diameter: 14 mm) affixed to the body [14], and two retro-reflective markers (diameter: 14 mm) affixed to the right and left extremities of the barbell [23], were collected by the Vicon T20 system (Vicon Motion Systems, Ltd., Oxford, UK), using 12 cameras operating at 250 Hz. The ground reaction force (GRF) was collected using a force platform (9287C, 0.9 m × 0.6 m; Kistler Instrumente AG, Winterthur, Switzerland) at 1000 Hz. We used two force platforms for the THPC and one for the OHPC. The maximum measured vertical ground reaction force of the force platforms was 20 kN. The kinematic data were smoothed using a fourth-order, low-pass Butterworth filter with optimal cut-off frequencies of 7.5 Hz and 15.0 Hz. These data were time-synchronized using Vicon Nexus software (Nexus 2, Vicon Motion Systems, Ltd., Oxford, UK). The kinetics data of the dominant leg were used for the data analyses.

The velocity of the center of gravity of the subject–bar system was calculated by numerically integrating the vertical displacement of the center of gravity of the system. The center of mass and the inertial parameters were estimated based on the body-segment parameters of Japanese athletes [24]. To compare the values per leg for both HPCs, the net GRF was calculated using the vertical GRF per leg minus half of the weight of the subject–bar system in the THPC. In the OHPC, the net GRF was calculated using the vertical GRF per leg minus the weight of the subject–bar system. The pull phase was defined as the minimum value of the vertical GRF during the initial position, to less than 10 N of the vertical GRF during the pull movement. Peak force was the maximum value of the vertical component of the net GRF during the pull phase. Power was calculated as the vertical GRF × vertical velocity of the center of gravity for the subject–bar system [1], and peak power was the maximum power during the pull phase. Instantaneous RFD was calculated by dividing the difference between the current and past vertical GRF by the elapsed time (0.001 s), and the peak RFD was the maximum value of the instantaneous RFD during the concentric phase [16].

### 2.4. Statistical Analyses

The intraclass correlation coefficients (ICCs) were calculated to determine the test–retest reliability of the measured variables. The normality of the data was assessed using the Shapiro–Wilk test. After normality was confirmed, a two-way (exercise × load) analysis of variance (ANOVA) with repeated measures was used to determine the difference between peak force, peak power, and peak RFD during both HPCs. When significant F-values were found, paired comparisons were used in a Bonferroni post hoc analysis to determine the significant differences. Effect sizes were calculated using Cohen’s *d* [25] and interpreted using the following scale: less than 0.2, trivial; between 0.2 and 0.5, small; between 0.5 and 0.8, medium; between 0.8 and 1.3, large; greater than 1.3, very large [26]. The alpha level was set at 0.05. All data are presented as mean ± SD. Statistical analyses were performed using SPSS (version 25, IBM Corp., Armonk, NY, USA).

## 3. Results

**Figure 2:**
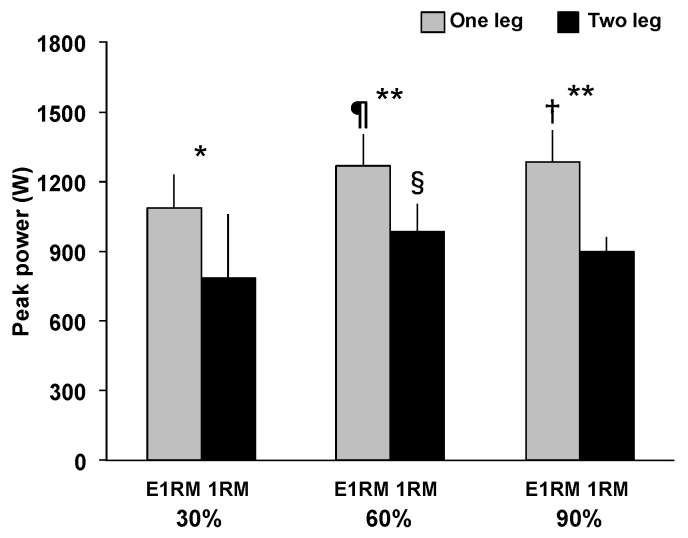
Peak power during the pull phase of a one- and two-leg hang power clean. *: Greater than the two-leg condition (*p* < 0.01). **: Greater than the two-leg condition (*p* < 0.001). †: Greater than 30% 1RM (*p* < 0.05). ¶: Greater than 30% 1RM (*p* < 0.01). §: Greater than 90% 1RM (*p* < 0.05). E1RM, estimated one-repetition maximum; 1RM, one-repetition maximum.

ICCs of peak power, peak force, and peak RFD for the OHPC and THPC were 0.80–0.99 and 0.80–0.97, respectively. Peak power (Figure 2) had no significant interaction, but a significant main effect for exercise (F = 47.03, *p* < 0.001, *η*^2^ = 0.85) and load (F = 9.42, *p* < 0.01, *η*^2^ = 0.54) was observed. Peak power during the OHPC was significantly greater than during the THPC at 30% (1087.46 ± 142.80 W vs. 786.75 ± 273.66 W, *p* < 0.01, *d* = 1.38), 60% (1270.17 ± 135.55 W vs. 987.59 ± 115.44 W, *p* < 0.001, *d* = 2.24), and 90% (1285.43 ± 134.24 W vs. 899.61 ± 62.43 W, *p* < 0.001, *d* = 3.69). Furthermore, peak power at 60% (1270.17 ± 135.55 W vs. 1087.46 ± 142.80 W, *p* < 0.01, *d* = 1.31) and 90% (1285.43 ± 134.24 W vs. 1087.46 ± 142.80 W, *p* < 0.05, *d* = 1.43) was significantly greater than at 30% during the OHPC, and peak power at 90% (899.61 ± 62.43 W vs. 987.59 ± 115.44 W, *p* < 0.05, *d* = 0.95) was significantly lower than at 60%, during the THPC.

**Figure 3:**
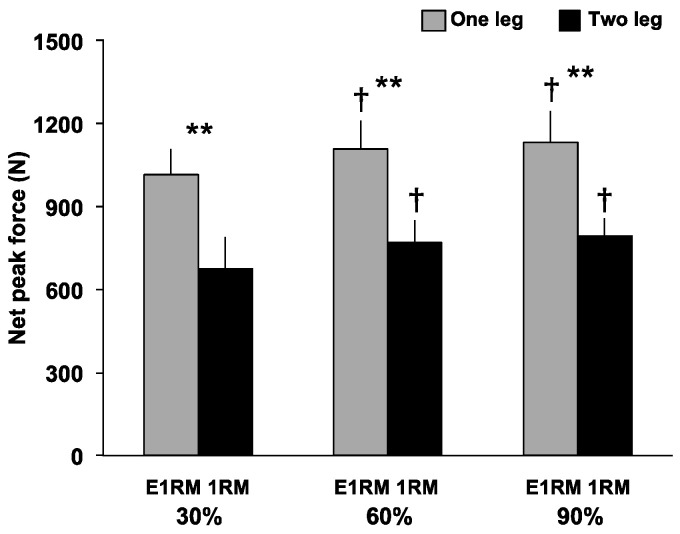
Peak force during the pull phase of a one- and two-leg hang power clean. Peak force during the pull phase of one- and two-leg hang power clean. **: Greater than the two-leg condition (*p* < 0.001). †: Greater than 30% 1RM (*p* < 0.05). E1RM, estimated one-repetition maximum; 1RM, one-repetition maximum.

Peak force (Figure 3) had no significant interaction, but a significant main effect for exercise (F = 93.76, *p* < 0.001, *η*^2^ = 0.92) and load (F = 12.40, *p* < 0.001, *η*^2^ = 0.61) was observed. The peak force of the OHPC was significantly greater than THPC at 30% (1014.91 ± 91.90 N vs. 673.19 ± 118.84 N, *p* < 0.001, *d* = 3.22), 60% (1108.89 ± 103.02 N vs. 771.29 ± 79.77 N, *p* < 0.001, *d* = 3.66), and 90% (1133.76 ± 109.98 N vs. 793.21 ± 64.43 N, *p* < 0.001, *d* = 3.78). Peak force at 60% (OHPC = 1108.89 ± 103.02 N vs. 1014.91 ± 91.90 N, *p* < 0.05, *d* = 0.96; THPC = 771.29 ± 79.77 N vs. 673.19 ± 118.84 N, *p* < 0.05, *d* = 0.97) and 90% (OHPC = 1133.76 ± 109.98 N vs. 1014.91 ± 91.90 N, *p* < 0.05, *d* = 1.17; THPC = 793.21 ± 64.43 N vs. 673.19 ± 118.84 N, *p* < 0.05, *d* = 1.26) was significantly greater than at 30%, during the OHPC and THPC.

**Figure 4:**
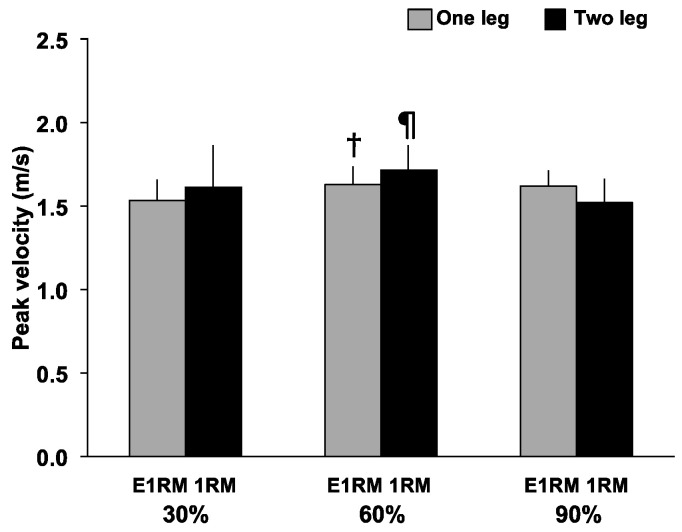
Peak velocity during the pull phase of a one- and two-leg hang power clean. Peak velocity during the pull phase of one- and two-leg hang power clean. †: Greater than 30% 1RM (*p* < 0.01). ¶: Greater than 90% 1RM (*p* < 0.01). E1RM, estimated one-repetition maximum; 1RM, one-repetition maximum.

Peak velocity (Figure 4) had a significant interaction (F = 4.11, *p* < 0.05, *η*^2^ = 0.34), and significant main effect for load (F = 3.80, *p* < 0.05, *η*^2^ = 0.32), but no significant main effect for exercise was observed. Peak velocity at 60% (1.63 ± 0.11 m/s vs. 1.54 ± 0.12 m/s, *p* < 0.01, *d* = 0.77) was significantly greater than at 30% during the OHPC, and the peak velocity at 60% (1.72 ± 0.15 m/s vs. 1.52 ± 0.14 m/s, *p* < 0.01, *d* = 1.33) was significantly greater than at 90% during the THPC.

**Figure 5:**
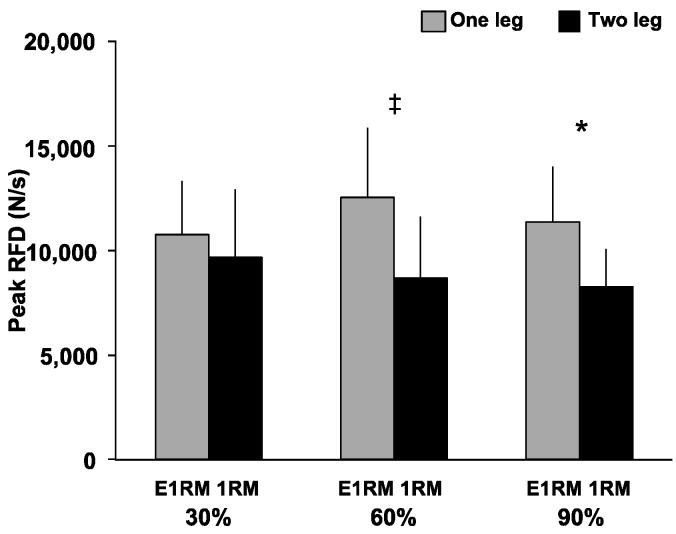
Peak rate of force development during the pull phase of one- and two-leg hang power clean. Peak velocity during the pull phase of one- and two-leg hang power clean. ‡: Greater than the two-leg condition (*p* < 0.05). *: Greater than the two-leg condition (*p* < 0.01). RFD, rate of force development; E1RM, estimated one-repetition maximum; 1RM, one-repetition maximum.

Peak RFD (Figure 5) had a significant interaction (F = 4.29, *p* < 0.05, *η*^2^ = 0.35), and significant main effect for exercise (F = 7.04, *p* < 0.05, *η*^2^ = 0.47), but no significant main effect for load was observed. Peak RFD during the OHPC was significantly greater than during the THPC at 60% (12,534.45 ± 3358.81 N/s vs. 8656.07 ± 2999.96 N/s, *p* < 0.05, *d* = 1.22) and 90% (11370.08 ± 2615.78 N/s vs. 8272.39 ± 1805.11 N/s, *p* < 0.01, *d* = 1.38).

## 4. Discussion

The main finding of this study was that the peak power, peak force, and peak RFD during the OHPC were greater than during the THPC, at loads of 60% and 90%. These results support our hypothesis that the kinetic data during the OHPC were greater than during the THPC.

To our knowledge, this is the first study to quantify the kinetics per leg during an OHPC and THPC using various loads. The kinetics data reported were lower than those of previous studies [1,4,6]. When the kinetics were calculated using the GRF in the THPC, peak power at 30%, 60%, and 90% 1RM were 2971.68 ± 387.06 W, 3708.22 ± 360.87 W, and 3854.34 ± 301.68 W, respectively. Thus, if the GRF of both legs is used for calculation, peak power of the THPC may be similar to the previous study (30%, 33.44 ± 7.53 W/kg; 60%, 43.87 ± 6.50 W/kg; and 90%, 43.76 ± 5.23 W/kg) [1].

Peak power, peak force, and peak RFD during the OHPC were significantly greater than the THPC. Moreover, the effect sizes of peak power and peak force at all loads were large (effect size > 1.38). Therefore, the OHPC may be a more explosive force- and power-developing exercise in the lower limbs when compared to the THPC.

A bilateral deficit occurs in jumping [10] and squat exercises [8,11], and it occurs more often in multi-joint exercises more than in single-joint exercises [27]. Therefore, it seems a bilateral deficit occurred in the HPC due to the multi-joint movement of the lower limbs and the kinetics of the OHPC being greater than the THPC. However, peak RFD at 30% showed no significant difference between the OHPC and THPC. Thus, a bilateral deficit may not affect peak RFD in the HPCs at 30%. Therefore, the OHPCs at 60% and 90% have the characteristics of explosive force and power development of the lower limbs, compared to the THPC, which may be affected by a bilateral deficit.

According to the changes in kinetics between the loads, peak power decreased at 90% compared to 60% during the THPC, but peak power was similar between these loads during the OHPC. Consequently, the characteristics of power output between the OHPC and THPC at 90% may be different. From the results of the analyses of peak force and peak velocity, it can be considered that greater peak power could occur at 90% because the OHPC exhibits a quick-lifting motion, even if the load increases. In addition, peak forces at 60% and 90% were greater than at 30% during both HPCs. These results concur with those stating peak forces of 45–80% 1RM in THPC are greater than at 30% 1RM [6]. Therefore, the OHPC exhibits greater power than the THPC even if the load increases, and the effect of the load on peak force is similar between both HPCs.

Peak RFD displayed significant differences between loads in both HPCs, as per previous studies [1,4]. Furthermore, there was only a significant difference between some loads at 30–85% 1RM [6]. Thus, peak RFD is more likely to be unaffected by load in the THPC [4]. Based on these reports, it seems that peak RFD with increased load is less likely to be affected in either HPC. Therefore, when using the HPC as a weightlifting exercise to improve the ability to exert high force in a short time, heavy loads may not be required.

Our results suggest that peak power, peak force, and peak RFD during the OHPC were greater than the THPC at loads of 60% and 90%. When a weightlifting exercise is performed with one leg, vertical jump height and relative power increase compared to two-leg exercises [28]. In addition, training adaptation is different between one- and two-leg exercises [29]. Therefore, coaches should prescribe the OHPC and THPC for athletes depending on the purpose for the training.

This study has several limitations. First, the sample size for this study was small, thus our findings may be more reliable with an increased sample size. Second, we did not measure the 1RM of the OHPC. When comparing the kinetics by measured 1RM, the 1RM of the OHPC may be more than half the load of the THPC. Therefore, the results of this study may differ. However, the kinetics of the loads up to 90% estimated 1RM during the OHPC (using half the barbell load at 90% 1RM of the THPC) obtained in this study may be similar to those obtained by measuring the 1RM of the OHPC. Additionally, the results of HPC cannot be applied to other clean exercises because they are performed in a variety of ways. If the start position of the clean exercise differs (from the floor, hanging, or mid-thigh), the characteristics of kinetics will differ [17]. Therefore, the kinetic characteristics of OHPC and THPC are expected to be different from those of other clean exercises.

## 5. Conclusions

This study indicates that the peak power, peak force, and peak RFD during the OHPC are greater than during the THPC with loads of 60% and 90%. Additionally, peak power decreased at 90% compared to 60% during the THPC, but not during the OHPC. These findings suggest that the OHPC at loads of 60% and 90% are weightlifting exercises that exhibit a greater explosive force and power development characteristics than the THPC. Therefore, these results are useful for strength and conditioning coaches when using OHPC as a weightlifting exercise to improve maximum power per leg.

## Data Availability

The data present in this study are available upon request from the corresponding author.
